# Controlled Formation of Silicon-Vacancy Centers in High-Pressure Nanodiamonds Produced from an “Adamantane + Detonation Nanodiamond” Mixture

**DOI:** 10.3390/nano14221843

**Published:** 2024-11-18

**Authors:** Dmitrii G. Pasternak, Rustem H. Bagramov, Alexey M. Romshin, Igor P. Zibrov, Vladimir P. Filonenko, Igor I. Vlasov

**Affiliations:** 1 Prokhorov General Physics Institute of the Russian Academy of Sciences, 38 Vavilov Str., Moscow 119991, Russia; romshin@nsc.gpi.ru; 2 Vereshchagin Institute of High-Pressure Physics of the Russian Academy of Sciences, 14 Kaluzhskoe Shosse, Troitsk, Moscow 108840, Russia; bagramov@hppi.troitsk.ru (R.H.B.); zibrov@hppi.troitsk.ru (I.P.Z.); filv@hppi.troitsk.ru (V.P.F.)

**Keywords:** nanodiamond, high-pressure synthesis, silicon-vacancy center, fluorescence, adamantane

## Abstract

Despite progress in the high-pressure synthesis of nanodiamonds from hydrocarbons, the problem of controlled formation of fluorescent impurity centers in them still remains unresolved. In our work, we explore the potential of a new precursor composition, a mixture of adamantane with detonation nanodiamond, both in the synthesis of nanodiamonds and in the controlled formation of negatively charged silicon-vacancy centers in such nanodiamonds. Using different adamantane/detonation nanodiamond weight ratios, a series of samples was synthesized at a pressure of 7.5 GPa in the temperature range of 1200–1500 °C. It was found that temperature around 1350 °C, is optimal for the high-yield synthesis of nanodiamonds <50 nm in size. For the first time, controlled formation of negatively charged silicon-vacancy centers in such small nanodiamonds was demonstrated by varying the atomic ratios of silicon/carbon in the precursor in the range of 0.01–1%.

## 1. Introduction

Advances of fluorescent nanodiamond (ND) applications in quantum photonics [1,2,3,4,5,6,7,8,9] and biomedicine technologies [10,11,12,13,14,15,16,17] initiate the development of methods for the mass production of fluorescent diamond particles of the smallest possible size. In particular, small ND sensors of temperature or magnetic fields are required for studying biological systems with high spatial resolution, down to the atomic level; small single photon ND emitters are in demand for efficient coupling with various types of nano-resonators in order to increase the photon emission rate. Currently, three main methods are used to produce NDs: ultrasonic milling (USM) of micron-sized diamonds using a ball mill [18,19,20,21], chemical vapor deposition (CVD) [18,22,23,24,25,26,27], and the high pressure–high temperature (HPHT) technique [16,28,29,30,31,32,33,34,35,36]. The appearance of structural defects in diamond during its mechanical crushing, as well as the impossibility of controlled formation of fluorescent centers in the resulting NDs, is characteristic of the USM method. CVD synthesis allows obtaining extremely small (micrograms) quantities of nanodiamonds in one deposition cycle. The most promising is the HPHT method, which allows obtaining 10–100 mg of nanodiamonds of high structural quality in one synthesis cycle.

Over the last 20 years, HPHT synthesis of micro- and nano-sized diamond particles from polycyclic aromatic hydrocarbons [28,29] and diamond-like molecules (adamantane) [30] at temperatures of 1300–1700 °C and a pressure of about 8–9 GPa has been successfully developing. The addition of halogen-containing components to the hydrocarbon precursor made it possible to reduce the synthesis temperature down to 700–900 °C [29,31]. In particular, a heterogeneous diamond powder consisting of particles both >1 μm and <100 nm in size was produced from a mixture of highly fluorinated graphite and naphthalene [29]. More uniform NDs with sizes below 50 nm were obtained using a single-component precursor, halogenated adamantane [31]. Recently, it has been shown that the addition of four nm detonation nanodiamond (DND) to naphthalene as a precursor, similar to the addition of halogen, significantly increases the yields of the nanosized diamond fraction in the HPHT synthesis product [16].

Despite progress in the high-pressure synthesis of nanodiamonds from hydrocarbons, the problem of controlled formation of fluorescent impurity centers in them still remains unresolved. To our knowledge, the possibility of controlled formation of fluorescent centers, in particular negatively charged “silicon-vacancy” (SiV^−^) and “germanium-vacancy” (GeV^−^), by varying the Si(Ge)/C relative content in the precursor over a wide range was previously studied only for micron-sized diamond particles synthesized from a mixture of adamantane and Si(Ge)-containing hydrocarbon [32]. The fluorescent properties of nanometer-sized diamonds (<100 nm), obtained both from purely hydrocarbon compounds and with the addition of halogens or DNDs to them, have not been studied over a wide range of doping element concentrations. To form SiV^−^ and GeV^−^ centers, high relative contents of the dopant element Si/C (Ge/C) in the precursor were used, ~1 at% and higher [16,29,33,34,36].

In this paper, we explore the potential of a new precursor composition, a mixture of adamantane with DND, both in the synthesis of NDs smaller than 50 nm in size and in the controlled formation of SiV^−^ centers in such NDs by varying the relative content of dopant silicon (Si/C) in the initial mixture in a wide range of 0.01–1% at.

## 2. Materials and Methods

Structural studies were performed for samples synthesized from a mixture of adamantane (C_10_H_16_, Aldrich, St. Louis, MO, USA, 99% purity) and DND (Adamas Nanotechnologies Inc., Raleigh, NC, USA, average sizes 3–4 nm), containing nitrogen impurity at a level of ~1 at% [37]. These two components were mixed in hexane in an agate mortar using ultrasound. Then, the mixture was pressed into a cylindrical tablet and placed in a graphite capsule inside a lithographic stone container of a toroidal-type apparatus [38]. The adamantane/DND weight ratios varied in the range 0/1–100/1. The samples were synthesized at a pressure of about 7.5 GPa in the temperature range of 1200–1500 °C for 20 s. The temperature inside the capsule was determined using a thermocouple with an accuracy of ±50 °C.

To form SiV^−^ centers in NDs, tetrakis(trimethylsilyl)silane (abbreviated further as tetrakis) C_12_H_36_Si_5_ (Sigma-Aldrich, St. Louis, MO, USA, >97%) was added to the initial adamantane/DND mixture in various proportions, varying the Si/C atomic ratio in the resulting mixture in the range of 1/100–1/10,000.

Additionally, samples were synthesized from a mixture of chloradamantane C_10_H_15_Cl (Sigma-Aldrich, 98%) and tetrakis, varying the Si/C atomic ratio in the resulting mixture also in the range of 1/100–1/10,000.

The sample phase composition and size of diamond crystallites were analyzed using an X-ray diffraction (XRD) Huber Imaging Plate Guinier camera G670 (Cu K_α1_ radiation, λ = 1.5406 Å, transmission mode, samples were applied to a Mylar film of 6 µm thickness), a JEOL 7001F Scanning Electron Microscope (SEM) and JEOL JEM-2100 Transmission Electron Microscope (TEM), JEOL, Tokyo, Japan. To estimate the size of diamond crystallites, the (220) diamond diffraction peaks were first approximated using the Lorentz profile, then the Full Width at Half Maximum of the peak maximum was measured, and the Scherrer formula was applied.

Photoluminescence (PL) and Raman spectra were measured at room temperature using a LabRam HR800 confocal spectrometer (Horiba Jobin-Yvon, Glasgow, UK). PL was excited with diode lasers at 473 nm and collected in backscattering geometry with an Olympus microscope objective (magnification ×50, numerical aperture NA = 0.55). Raman spectra were recorded with a spectral resolution of 0.2 cm^−1^ and a spatial resolution of about 1–2 μm. The laser power reaching the diamond nanoparticles was ~0.1 mW.

## 3. Results and Discussion

### 3.1. Characterization of NDs Synthesized from Adamantane/DND Mixture

At the first stage of this research, we (1) optimized adamantane/DND weight ratio in the range of 1/1–100/1 for high-yield synthesis of NDs and (2) determined the dependence of their characteristic sizes on the synthesis temperature. The synthesis was carried out at three different temperatures in the range of 1200–1500 °C and a pressure of about 7.5 GPa.

**Figure 1:**
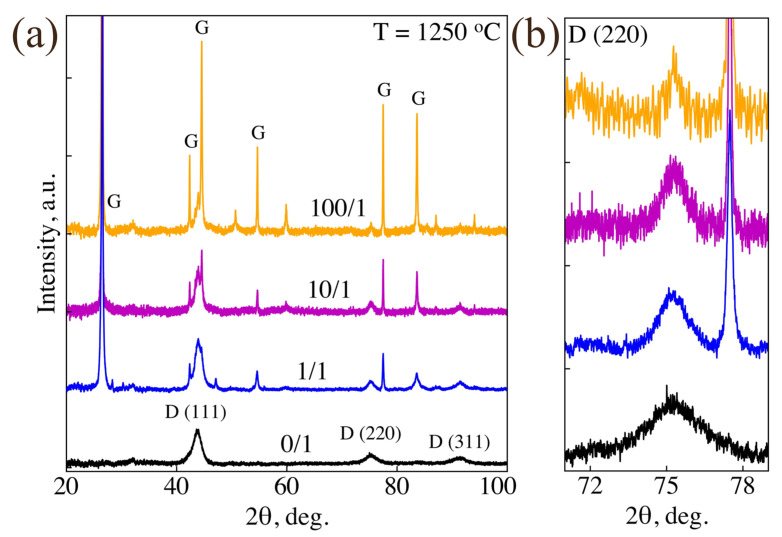
(**a**) XRD patterns of four different HPHT samples synthesized at 1250 °C from adamantane/DND mixture in the following weight proportions: 0/1, 1/1, 10/1 and 100/1. For convenience of comparative analysis, the diffraction patterns are normalized to the intensity of the (220) diamond diffraction peak, and the background associated with the scattering of X-rays on the substrate is subtracted from them. D—diamond, G—graphite. (**b**) Zoomed part of XRD pattern around (220) diamond diffraction peak.

Optimization of adamantane/DND weight ratio in the precursor was carried out at a synthesis temperature of 1250 ± 50 °C. Figure 1 shows the XRD patterns of four samples obtained from DND without the addition of adamantane, as well as from adamantane/DND mixtures prepared in the weight ratios of 1/1, 10/1, and 100/1. These samples will be denoted hereinafter as “0/1”, “1/1”, “10/1” and “100/1”, respectively. The XRD patterns of the samples synthesized with adamantane contain both diamond and graphite diffraction peaks. One can see that the graphite/diamond weight ratio in the samples increases with the amount of adamantane in the precursor. In addition, the size of diamond crystallites increases with the adamantane amount, as evidenced by the decrease in the width of the (220) diamond diffraction peak (Figure 1b). According to estimates using the Scherrer formula, the average diameters of diamond crystallites in the samples “0/1”, “1/1” and “10/1” are 5 nm, 8 nm and 11 nm, respectively.

**Figure 2:**
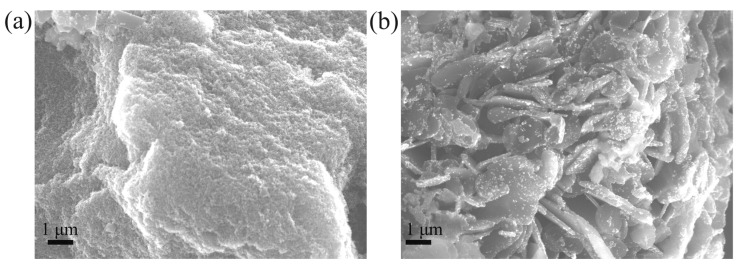
(**a**) SEM images of samples synthesized at 1250 °C from adamantane/DND mixture in weight proportion 10/1 (**a**) and 100/1 (**b**).

Figure 2 shows typical SEM images of samples “10/1” and “100/1” taken from the central part of the graphite capsule. SEM images shown in Figure 2a,b are typical, respectively, for massifs of nanometer-sized diamond particles and micron-sized graphite plates with diamond nanoparticles embedded. The interpretation of these images is confirmed by the Raman spectra recorded for the sample areas shown in Figure 2a,b. Thus, with an increase in the adamantane/DND weight ratio from 10/1 to 100/1, the amount of graphite in the samples increases significantly. It can be assumed that in the sample “10/1”, adamantane actively participates in the growth of the initial DNDs; according to the XRD analysis (Figure 1), their size changes from 5 nm to 11 nm. Meanwhile, in the sample “100/1”, adamantane mainly transfers into graphite, as evidenced by the significant increase in the graphite/diamond peak intensity ratio in the XRD pattern of this sample compared to the sample “10/1”. In order to have as-grown samples with a high yield of nanodiamonds, we further reduced the range of the studied adamantane/DND weight ratios to 1/1–25/1.

**Figure 3:**
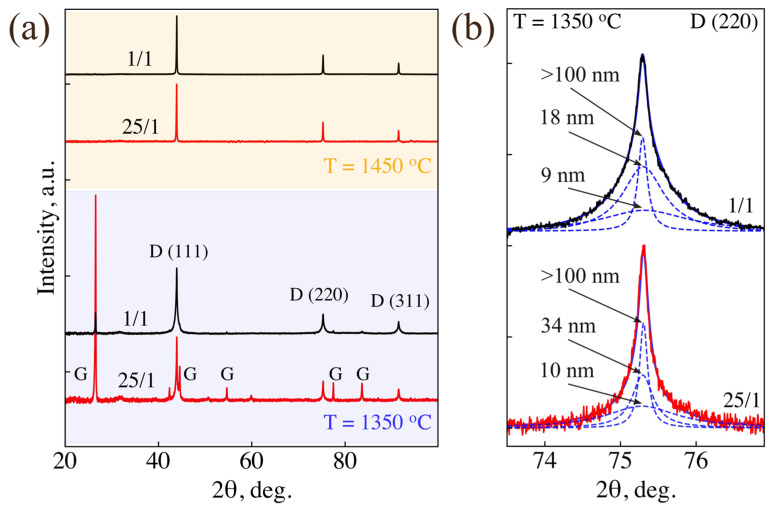
(**a**) Wide-angle XRD patterns of “1/1” (black) and “25/1” (red) samples synthesized at 1350 and 1450 °C. (**b**) Approximation of (220) diamond diffraction peaks by Lorentz profiles for samples “1/1” and “25/1” synthesized at 1350 °C. The peak shape is most accurately fitted by the superposition of three profiles. The arrows indicate the average sizes of diamond nanoparticles estimated for each profile.

Figure 3a shows the XRD patterns of samples “1/1” and “25/1” synthesized at 1350 ± 50 and 1450 ± 50 °C. In both samples the graphite/diamond peak intensity ratio at 1350 °C is significantly less than at 1250 °C (Figure 1), and at 1450 °C graphite is not detected at all by XRD. At 1350 °C, the (220) diamond diffraction peaks are most accurately fitted by the superposition of three Lorentz profiles (Figure 3b). The widths of these three profiles correspond to the next average sizes of diamond crystallites: 9, 18, ≥100 nm for the sample “1/1” and 10, 34, ≥100 nm for the sample “25/1”. At the synthesis temperature of 1450 °C, the diffraction peaks narrow to values close to their instrumental broadening, when the Scherrer formula becomes inapplicable. In that case, the characteristic sizes of crystallites, lying in the range of 100–300 nm for both samples, were determined based on SEM data.

**Figure 4:**
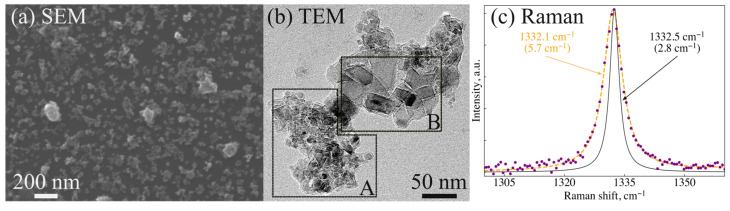
SEM, TEM images and Raman spectrum of the sample “25/1” synthesized at 1350 °C. (**a**) SEM image of diamond nanoparticles dispersed on a substrate from alcohol suspension. (**b**) TEM image of the ND agglomerate prepared by drying a drop of the suspension on a grid. The characteristic sizes of diamond crystallites are less than 10 nm in the marked area A, and 20–50 nm in the area B. (**c**) Typical Raman spectrum of the sample. The diamond peak (orange dotted line) is shifted to the position of 1332.1 cm^−^^1^ and broadened up to 5.7 cm^−1^ relative to the diamond peak (black line) recorded for the natural bulk IIa diamond.

The assessment of sample sizes using XRD is also confirmed by SEM, TEM and Raman spectroscopy. Figure 4 shows the results of characterization of sample “25/1” obtained at 1350 °C. The part of the sample taken from a center of the graphite capsule was analyzed. The SEM image (Figure 4a) shows ND particles dispersed from an alcohol suspension onto a silicon substrate. Particles with characteristic sizes smaller than 50 nm predominate. TEM analysis revealed peculiarities in the size distribution (Figure 4b). This distribution shows two characteristic sizes: below 10 nm, which is close to the initial size (Figure 4b, region A); and 20–50 nm (Figure 4b, region B). We explain this by the existence in the initial DND of both discrete diamond crystallites and their stable aggregates [39]. Mixing of discrete crystallites with adamantane results in their active growth during the synthesis. No growth of DND crystallites located inside the aggregate occurs due to the absence of their interaction with adamantane (see next paragraph for details). Note that a similar result (10 and 34 nm) was obtained for sample “25/1” by fitting the (220) diamond peak (1 and 2 components in Figure 3b). At the same time, nanodiamonds ≥ 100 nm were observed at the sample periphery in SEM. This corresponds to the third fit component of the (220) diamond peak of sample “25/1” (Figure 3b). The increase in the ND sizes from the sample center to its periphery is associated with higher temperature near the walls of the graphite heater. Raman spectra recorded for a number of ND clusters of the sample “25/1” are characterized by a shift of the diamond line relative to the standard position (1332.5 cm^−^^1^) by 0.4 cm^−^^1^ towards lower frequencies, broadening up to 6 cm^−^^1^ and a slight asymmetric deviation from the Lorentzian profile (Figure 4c). All these features are explained by the phonon confinement effect [40]. According to the experimental results and calculations based on the phonon-confinement model [40,41], such a shift of the diamond line is characteristic of particles with sizes below 50 nm. Thus, the estimates of the characteristic sizes of ND for sample “25/1” obtained by XRD, TEM and Raman spectroscopy are in good agreement with each other. It should also be noted that graphite inclusions in this sample are extremely rare, they are easily detected even with an optical microscope and reveal themselves in the Raman spectrum as two wide bands at frequencies of about 1350 cm^−^^1^ and 1580 cm^−^^1^. Based on the Raman analysis, we come to the conclusion of the high yield of nanodiamond fraction in the sample “25/1” synthesized at 1350 °C.

Based on the structural analysis of the synthesized samples, we offer the following interpretation of the diamond crystallite formation from the adamantane/DND mixture at a synthesis temperature of 1200–1500 °C and a pressure of about 7.5 GPa. At these p–T parameters, thermal decomposition of both adamantane molecules and, partially, DND crystallites occurs with the formation of a hydrocarbon fluid. The smallest DND crystallites decompose, while larger ones remain in the reaction mixture. Additional experiments on the thermobaric treatment of DND in the temperature range of 1200–1500 °C confirm the fact of decomposition of the smallest nanodiamonds into amorphous carbon, leading to an increase in the average size of the surviving diamond nanoparticles (for more details, see Appendix A and Appendix B). Similar results have been obtained in work [42] under annealing DNDs at atmospheric pressure. Thus, larger and more perfect DND crystallites act as nuclei that initiate diamond growth from the surrounding hydrocarbon fluid. In those areas of the fluid that contain few or no nuclei, carbon crystallizes predominantly into graphite, similar to what happens during the thermobaric treatment of pure adamantane under similar conditions. In samples with a lower initial adamantane fraction, there are fewer such areas, and less graphite is formed (Figure 1). As follows from the XRD data, at lower temperatures, 1250 °C, the rates of diamond formation and graphitization compete with each other. As a result, samples are obtained that consist of a mixture of ND and graphite (Figure 1). At a higher temperature (1450 °C), the diamond formation is dominated (Figure 3).

The obtained results indicate the potential of using adamantane in combination with DND for the synthesis of diamond nanoparticles (<100 nm) in the temperature range of 1200–1400 °C and pressures of 7.5 GPa. Note that in this p–T range, adamantane transforms into graphite [30], whereas DND partially amorphizes (see Appendix B). Until now, the problem of stable synthesis of NDs smaller than 100 nm in size from hydrocarbons at pressures <8 GPa and temperatures <1400 °C was solved by adding halogen-containing compounds to the precursor [31]. In our work, stable synthesis of NDs at such “soft” p–T parameters was achieved by adding small diamond nuclei to adamantane.

### 3.2. The Controlled Formation of SiV^−^ Centers in NDs

In the following experiments, the 1/1 and 25/1 adamantane/DND compositions with the addition of tetrakis containing silicon were used to synthesize samples at the temperature of 1350 °C and the pressure of 7.5 GPa (see Section 2 for details).

**Figure 5:**
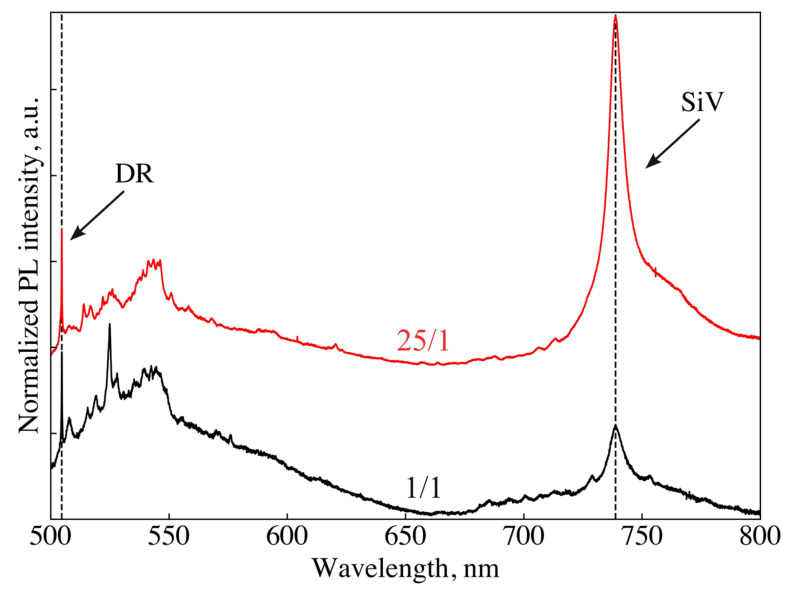
PL spectra of the samples synthesized at 1350 °C from adamantane/DND/tetrakis mixture at adamantane/DND weight proportions 25/1 (red) and 1/1 (black), and Si/C atomic ratio 1%. The spectra are normalized to the intensity of the diamond Raman (DR) line. Zero-phonon line of SiV^−^ center and DR line are observed at 738.5 nm and 504.8 nm, respectively. “Palisade” of narrow lines observed throughout the range 500–800 nm is typical for N-doped H-terminated NDs [43].

A series of PL spectra were measured for different ND clusters of “25/1” and ”1/1” samples produced at a ratio of Si/C = 1/100 at. In most of the spectra, the diamond Raman line had a width ≥5.7 cm^−1^ and a position of ≤1332.0 cm^−1^. This suggests that the ND clusters under study contain nanoparticles with a size <50 nm. Figure 5 shows typical PL spectra of two samples, “25/1” (red) and “1/1” (black). In both spectra, in addition to the zero-phonon line (ZPL) of SiV^−^ fluorescence at 738 nm, a large number of narrow, discrete and overlapping lines are observed in the range between 500 and 800 nm. We associate these lines with the radiative recombination between nitrogen impurities and acceptors on the hydrogen-terminated surface of diamond nanoparticles [43]. As shown in Figure 5, the integrated intensity of the SiV^−^ ZPL is five times higher in sample “25/1” as compared to the sample “1/1”. We attribute this difference to the suppression of SiV^−^ luminescence in the “1/1” sample due to the high concentration (~1 at%, see Section 2) of nitrogen impurities in the DND component of the precursor. The quenching of fluorescence at a defect concentration higher than 10^−^^3^ in inorganic crystals is explained by dipole–quadrupole interactions [44]. For instance, the fluorescence quenching for different color centers at nitrogen impurity concentrations above 1000 ppm (in the precursor) has been observed in both natural [45] and CVD diamonds [46]. Another possible reason for stronger SiV^−^ fluorescence in sample “25/1” could be the larger average ND sizes as compared to sample “1/1” (see Figure 3b) As the size of diamond nanoparticles increases, the probability of fluorescence quenching due to SiV^−^ interaction with acceptors on hydrogen-terminated diamond surfaces decreases. This effect will be discussed in more detail below.

Based on the comparison of the PL spectra of the “1/1” and “25/1” samples, for further studies of the controlled formation of SiV^−^ centers in NDs <50 nm in size, the 25/1 adamantane/DND composition was chosen as initial.

**Figure 6:**
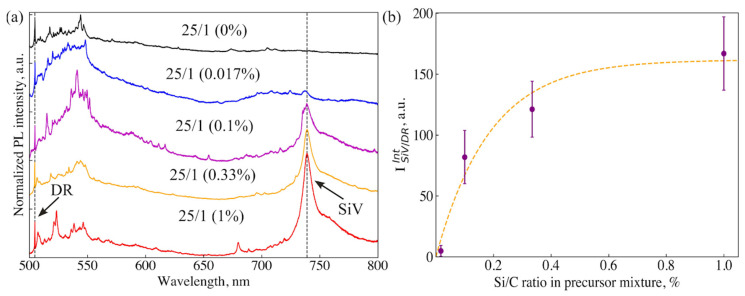
(**a**) PL spectra of the sample “25/1” synthesized at 1350 °C from adamantane/DND/tetrakis mixture with atomic ratio Si/C varying from 0% to 1%. The spectra are normalized to the intensity of the diamond Raman (DR) line. (**b**) The dependence of the integrated fluorescence intensity SiV^−^ normalized to the DR line (I^int^_SiV/DR_) on the atomic ratio Si/C in the precursor. The statistical error in determining I^int^_SiV/DR_/DR was calculated based on averaging ten PL spectra for each of the five samples.

Next, five ND samples synthesized at 1350 °C from a mixture of adamantane/DND/tetrakis with different Si/C ratios in the precursor, such as 0, 0.017, 0.1, 0.33, 1% (at.), were analyzed: 0, 0.017, 0.1, 0.33, 1% (at.). From here on, we will designate these samples as “25/1” (0%), “25/1” (0.17%), etc. Characteristic PL spectra of these samples are shown in Figure 6a. The set of lines in them is similar to those observed in the spectra in Figure 5.

Figure 6b shows the dependence of the normalized integrated intensity of SiV^−^ fluorescence (I^int^_SiV/DR_) on the Si/C atomic ratio. SiV^−^ fluorescence continuously increases with increasing silicon in the range of 0–0.5% and reaches a plateau with I^int^_SiV/DR_ = 165 ± 10 at higher concentrations. Analysis of more than fifty PL spectra shows that the position and width of the SiV^−^ line fluctuates within small ranges, 738.5 ± 0.5 nm and 10 ± 2 nm, respectively, from spectrum to spectrum regardless of the Si/C ratio. We associate such fluctuations with the heterogeneity of the structural quality of diamond crystallites, in particular, with the irregular distribution of nitrogen in the samples.

Let us point out one of the possible ways to increase the intensity of SiV^−^ fluorescence in ND synthesized from the adamantane/DND/tetrakis mixture. The surface of such NDs is terminated with hydrogen, which is known to induce free holes in the diamond surface layer [47]. The interaction of SiV^−^ centers with these holes can lead to a change in the charge state of the centers and thus to the quenching of SiV^−^ fluorescence. Since SiV^−^ centers are distributed in the diamond volume and holes are localized near its surface, the probability of interaction between them will increase with decreasing dimensions of the diamond nanoparticle. Thus, the removal of hydrogen and associated holes from the ND surface may result in increased intensity of SiV^−^ fluorescence. Our investigation of surface hydrogen effect on the SiV^−^ fluorescence intensity depending on the size of diamond nanoparticles is in progress.

**Figure 7:**
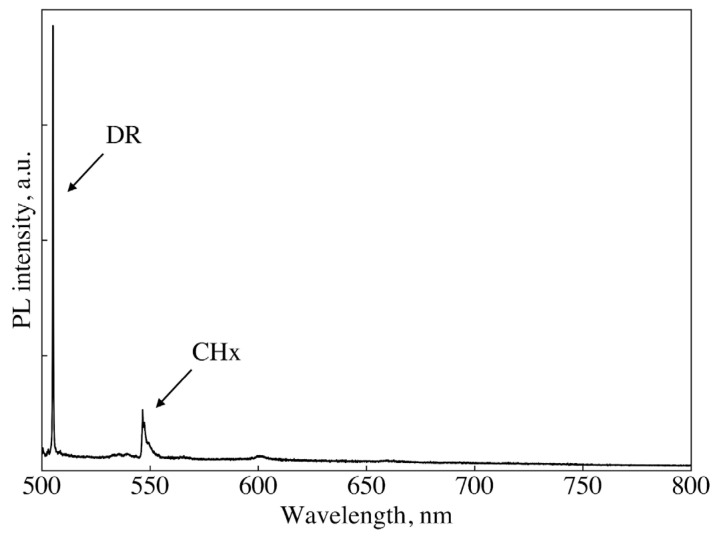
Raman spectrum of the sample synthesized at 1350 °C from chloradamantane/tetrakis mixture with atomic ratio Si/C = 1/1000. Only the lines of DR (504.8 nm) and the stretching vibrations of surface CHx groups (near 550 nm) are observed in the spectrum.

In the Introduction, it was noted that the previously proposed approach to the mass synthesis of 1–100 nm NDs using halogen-containing organic precursors does not allow for a controllable SiV^−^ formation in such NDs. To experimentally verify the negative effect of halogens on Si doping, samples were synthesized from chloroadamantane with the addition of tetrakis (Si/C atomic ratio 1/100–1/10,000). Samples were synthesized at 1350 °C and 7.5 GPa. Regardless of the Si/C ratio, the SiV^−^ fluorescence was not observed in these samples. There are only the Raman line of diamond (504.8 nm) and stretching vibrations of surface CHx groups (near 550 nm) in the PL spectrum typical for these samples (Figure 7). The ratio between the intensities of the Raman lines of diamond and CHx ~10 indicates that the sizes of ND in the sample are in the range of 20–50 nm [35].

We believe that chlorine bind silicon to the highly volatile compound SiCl_4_ during synthesis, preventing the formation of SiV^−^ centers in diamonds. Indeed, the bond dissociation energy (BDE) of Si-Cl in this compound is quite high, 462 kJ/mol [48]. For comparison, the BDE of C-Cl in the compound CCl_4_ is significantly lower, 296 kJ/mol. Note that the BDE of Si-F in the compound SiF_4_ is even higher, 697 kJ/mol. Perhaps due to the effective binding of silicon with fluorine, the authors of works [16,28,29,36] on the synthesis of ND from fluorinated hydrocarbons use atomic ratios of Si/F~1 in the precursor to form.

## 4. Conclusions

A number of samples from a new combination of compounds in the precursor, adamantane and DND, were synthesized by the HPHT technique at a pressure of about 7.5 GPa in the temperature range of 1200–1500 °C. It was found that at the adamantane/DND weight ratio of 1/1 and 25/1 and synthesis temperatures of 1200–1400 °C, the diamond phase dominates in the obtained samples and the characteristic sizes of diamond nanoparticles are in the range of 10–50 nm.

For the first time, the possibility of controlled formation of SiV^−^ centers in HPHT NDs smaller than 50 nm in size was demonstrated by varying the atomic ratios of silicon/carbon in the precursor from 1/100 to 1/10,000. It should be noted that all spectral and structural investigations were conducted on the as-synthesized samples, without any special pretreatment, which indicates the high yield of nanodiamonds in the samples. Until now, to demonstrate SiV^−^ fluorescence in small (<50 nm) NDs, produced samples were chemically purified and separated by size [16,29,33]. The possibility of controlled formation of SiV^−^ centers in synthesized ND particles makes it possible to produce on their base both sources of single photons containing only one SiV^−^ emitter in an individual particle, and nanosensors of ultra-local temperature fields, containing large ensembles of SiV^−^ emitters in one particle. Eventually, the stable HPHT synthesis of a large amount (~10 mg per synthesis cycle) of fluorescent NDs smaller than 50 nm proposed in this work opens up opportunities for their various technological applications.

## Figures and Tables

**Figure A1:**
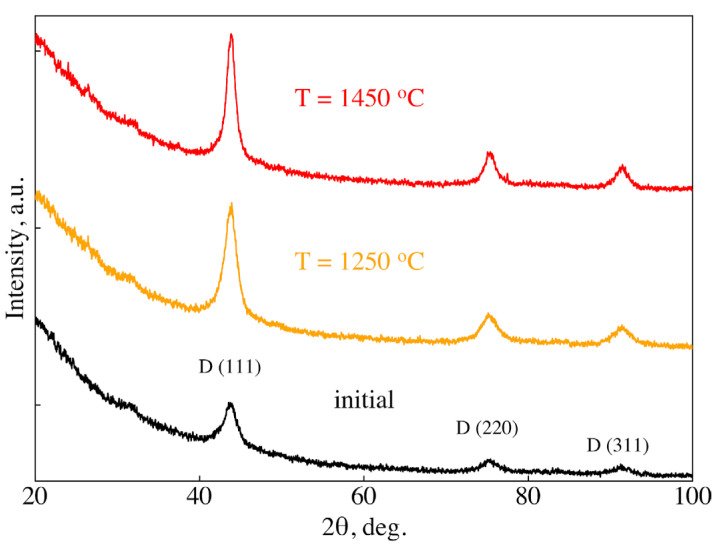
XRD pattern of three samples: initial (black), treated at 7.5 GPa pressure and temperatures of 1250 °C (orange) and 1450 °C (red). Only diffraction peaks of diamond are observed in all three samples. The estimation of the crystallite sizes according to the Scherrer formula indicates that the average size of diamond crystallites increases from 4–5 nm (initial) to 7 nm (1450 °C). Note that amorphous carbon, which dominates in the Raman spectra (see below), is not detected by the XRD.

**Figure A2:**
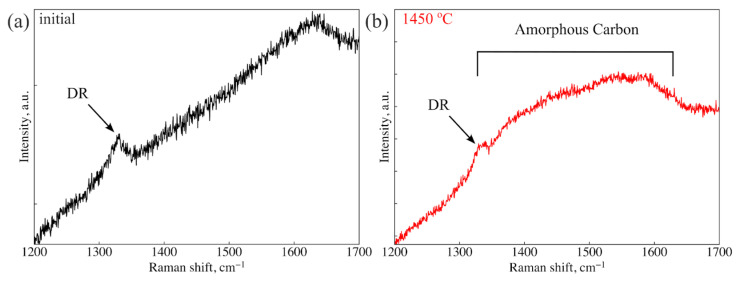
(**a**) The typical Raman spectrum of the untreated DND consists of an asymmetric diamond peak (DR) at 1328.5 cm^−1^ and a band at 1630 cm^−1^ that overlap with the initial fragment of broadband (500–700 nm) fluorescence background [37]. (**b**) Typical Raman spectrum of DND treated at 1450 °C is characterized by a broad band of amorphous carbon (1300–1600 cm^−1^), which dominates the spectrum [49]; a diamond (DR) peak is barely distinguished against the fluorescence background. Similar Raman spectra were observed for DND treated at 1250 °C.

## Data Availability

Data are contained within the article.

## References

[1] Kurtsiefer C., Mayer S., Zarda P., Weinfurter H. (2000). Stable Solid-State Source of Single Photons. Phys. Rev. Lett..

[2] Le Floch J.-M., Bradac C., Nand N., Castelletto S., Tobar M.E., Volz T. (2014). Addressing a Single Spin in Diamond with a Macroscopic Dielectric Microwave Cavity. Appl. Phys. Lett..

[3] Lončar M., Faraon A. (2013). Quantum Photonic Networks in Diamond. MRS Bull..

[4] Ermakova A., Pramanik G., Cai J.-M., Algara-Siller G., Kaiser U., Weil T., Tzeng Y.-K., Chang H.C., McGuinness L.P., Plenio M.B. (2013). Detection of a Few Metallo-Protein Molecules Using Color Centers in Nanodiamonds. Nano Lett..

[5] Bernien H., Hensen B., Pfaff W., Koolstra G., Blok M.S., Robledo L., Taminiau T.H., Markham M., Twitchen D.J., Childress L. (2013). Heralded Entanglement between Solid-State Qubits Separated by Three Metres. Nature.

[6] Benedikter J., Kaupp H., Hümmer T., Liang Y., Bommer A., Becher C., Krueger A., Smith J.M., Hänsch T.W., Hunger D. (2017). Cavity-Enhanced Single-Photon Source Based on the Silicon-Vacancy Center in Diamond. Phys. Rev. Appl..

[7] Ngan K., Zhan Y., Dory C., Vučković J., Sun S. (2023). Quantum Photonic Circuits Integrated with Color Centers in Designer Nanodiamonds. Nano Lett..

[8] Zuber J.A., Li M., Puigibert M.G., Happacher J., Reiser P., Shields B.J., Maletinsky P. (2023). Shallow Silicon Vacancy Centers with Lifetime-Limited Optical Linewidths in Diamond Nanostructures. Nano Lett..

[9] Klotz M., Waltrich R., Lettner N., Agafonov V.N., Kubanek A. (2024). Strongly Coupled Spins of Silicon-Vacancy Centers inside a Nanodiamond with Sub-Megahertz Linewidth. Nanophotonics.

[10] Fu C.-C., Lee H.-Y., Chen K., Lim T.-S., Wu H.-Y., Lin P.-K., Wei P.-K., Tsao P.-H., Chang H.-C., Fann W. (2007). Characterization and Application of Single Fluorescent Nanodiamonds as Cellular Biomarkers. Proc. Natl. Acad. Sci. USA.

[11] Romshin A.M., Zeeb V., Martyanov A.K., Kudryavtsev O.S., Pasternak D.G., Sedov V.S., Ralchenko V.G., Sinogeykin A.G., Vlasov I.I. (2021). A new approach to precise mapping of local temperature fields in submicrometer aqueous volumes. Sci. Rep..

[12] Tisler J., Reuter R., Lämmle A., Jelezko F., Balasubramanian G., Hemmer P.R., Reinhard F., Wrachtrup J. (2011). Highly Efficient FRET from a Single Nitrogen-Vacancy Center in Nanodiamonds to a Single Organic Molecule. ACS Nano.

[13] Alkahtani M. (2023). Silicon Vacancy in Boron-Doped Nanodiamonds for Optical Temperature Sensing. Materials.

[14] Kromka A., Varga M., Aubrechtová Dragounová K., Babčenko O., Pfeifer R., Flatae A.M., Sledz F., Akther F., Agio M., Potocký Š. (2024). High-Yield Production of SiV-Doped Nanodiamonds for Spectroscopy and Sensing Applications. ACS Appl. Nano Mater..

[15] Fujiwara M., Uchida G., Ohki I., Liu M., Tsurui A., Yoshikawa T., Nishikawa M., Mizuochi N. (2022). All-Optical Nanoscale Thermometry Based on Silicon-Vacancy Centers in Detonation Nanodiamonds. Carbon.

[16] Weil T., Liu W., Alam M.N.A., Liu Y., Agafonov V.N., Qi H., Koynov K., Davydov V.A., Uzbekov R., Kaiser U. (2022). Silicon-Vacancy Nanodiamonds as High Performance Near-Infrared Emitters for Live-Cell Dual-Color Imaging and Thermometry. Nano Lett..

[17] Wu Y., Weil T. (2022). Recent Developments of Nanodiamond Quantum Sensors for Biological Applications. Adv. Sci..

[18] Neu E., Arend C., Gross E., Guldner F., Hepp C., Steinmetz D., Zscherpel E., Ghodbane S., Sternschulte H., Steinmüller-Nethl D. (2011). Narrowband Fluorescent Nanodiamonds Produced from Chemical Vapor Deposition Films. Appl. Phys. Lett..

[19] Lindner S., Bommer A., Muzha A., Krueger A., Gines L., Mandal S., Williams O., Londero E., Gali A., Becher C. (2018). Strongly Inhomogeneous Distribution of Spectral Properties of Silicon-Vacancy Color Centers in Nanodiamonds. New J. Phys..

[20] Chang S.L.Y., Reineck P., Krueger A., Mochalin V.N. (2022). Ultrasmall Nanodiamonds: Perspectives and Questions. ACS Nano.

[21] Liang Y., Ozawa M., Krueger A. (2009). A General Procedure to Functionalize Agglomerating Nanoparticles Demonstrated on Nanodiamond. ACS Nano.

[22] Neu E., Steinmetz D., Riedrich-Möller J., Gsell S., Fischer M., Schreck M., Becher C. (2011). Single Photon Emission from Silicon-Vacancy Colour Centres in Chemical Vapour Deposition Nano-Diamonds on Iridium. New J. Phys..

[23] Neu E., Fischer M., Gsell S., Schreck M., Becher C. (2011). Fluorescence and Polarization Spectroscopy of Single Silicon Vacancy Centers in Heteroepitaxial Nanodiamonds on Iridium. Phys. Rev. B.

[24] Neu E., Hepp C., Hauschild M., Gsell S., Fischer M., Sternschulte H., Steinmüller-Nethl D., Schreck M., Becher C. (2013). Low-Temperature Investigations of Single Silicon Vacancy Colour Centres in Diamond. New J. Phys..

[25] Becker J.N., Neu E. (2020). The Silicon Vacancy Center in Diamond. Semiconductors and Semimetals.

[26] Li K., Zhou Y., Rasmita A., Aharonovich I., Gao W.B. (2016). Nonblinking Emitters with Nearly Lifetime-Limited Linewidths in CVD Nanodiamonds. Phys. Rev. Appl..

[27] De Feudis M., Tallaire A., Nicolas L., Brinza O., Goldner P., Hétet G., Bénédic F., Achard J. (2020). Large-Scale Fabrication of Highly Emissive Nanodiamonds by Chemical Vapor Deposition with Controlled Doping by SiV and GeV Centers from a Solid Source. Adv Mater. Inter.

[28] Davydov V.A., Rakhmanina A.V., Agafonov V., Narymbetov B., Boudou J.-P., Szwarc H. (2004). Conversion of Polycyclic Aromatic Hydrocarbons to Graphite and Diamond at High Pressures. Carbon.

[29] Davydov V.A., Rakhmanina A.V., Lyapin S.G., Ilichev I.D., Boldyrev K.N., Shiryaev A.A., Agafonov V.N. (2014). Production of Nano- and Microdiamonds with Si-V and N-V Luminescent Centers at High Pressures in Systems Based on Mixtures of Hydrocarbon and Fluorocarbon Compounds. Jetp Lett..

[30] Ekimov E.A., Kudryavtsev O.S., Mordvinova N.E., Lebedev O.I., Vlasov I.I. (2018). High-Pressure Synthesis of Nanodiamonds from Adamantane: Myth or Reality?. ChemNanoMat.

[31] Ekimov E.A., Lyapin S.G., Grigoriev Y.V., Zibrov I.P., Kondrina K.M. (2019). Size-Controllable Synthesis of Ultrasmall Diamonds from Halogenated Adamantanes at High Static Pressure. Carbon.

[32] Ekimov E.A., Kondrin M.V., Krivobok V.S., Khomich A.A., Vlasov I.I., Khmelnitskiy R.A., Iwasaki T., Hatano M. (2019). Effect of Si, Ge and Sn Dopant Elements on Structure and Photoluminescence of Nano- and Microdiamonds Synthesized from Organic Compounds. Diam. Relat. Mater..

[33] Plakhotnik T., Duka T., Davydov V.A., Agafonov V.N. (2023). Formation of SiV Centers by Doping in Bottom-up Grown HPHT Nanodiamonds and Its Implication for Optical Nanosensig. Diam. Relat. Mater..

[34] Ekimov E.A., Kondrin M.V., Lyapin S.G., Grigoriev Y.V., Razgulov A.A., Krivobok V.S., Gierlotka S., Stelmakh S. (2020). High-Pressure Synthesis and Optical Properties of Nanodiamonds Obtained from Halogenated Adamantanes. Diam. Relat. Mater..

[35] Kudryavtsev O.S., Bagramov R.H., Pasternak D.G., Satanin A.M., Lebedev O.I., Filonenko V.P., Vlasov I.I. (2023). Raman Fingerprints of Ultrasmall Nanodiamonds Produced from Adamantane. Diam. Relat. Mater..

[36] Choi S., Leong V., Davydov V.A., Agafonov V.N., Cheong M.W.O., Kalashnikov D.A., Krivitsky L.A. (2018). Varying Temperature and Silicon Content in Nanodiamond Growth: Effects on Silicon-Vacancy Centres. Sci. Rep..

[37] Shenderova O.A., Vlasov I.I., Turner S., Van Tendeloo G., Orlinskii S.B., Shiryaev A.A., Khomich A.A., Sulyanov S.N., Jelezko F., Wrachtrup J. (2011). Nitrogen Control in Nanodiamond Produced by Detonation Shock-Wave-Assisted Synthesis. J. Phys. Chem. C.

[38] Khvostantsev L.G., Slesarev V.N., Brazhkin V.V. (2004). Toroid Type High-Pressure Device: History and Prospects. High Press. Res..

[39] Vlasov I.I., Shenderova O., Turner S., Lebedev O.I., Basov A.A., Sildos I., Rähn M., Shiryaev A.A., Van Tendeloo G. (2010). Nitrogen and Luminescent Nitrogen-Vacancy Defects in Detonation Nanodiamond. Small.

[40] Ager J.W., Veirs D.K., Rosenblatt G.M. (1991). Spatially Resolved Raman Studies of Diamond Films Grown by Chemical Vapor Deposition. Phys. Rev. B.

[41] Korepanov V.I., Hamaguchi H., Osawa E., Ermolenkov V., Lednev I.K., Etzold B.J.M., Levinson O., Zousman B., Epperla C.P., Chang H.-C. (2017). Carbon Structure in Nanodiamonds Elucidated from Raman Spectroscopy. Carbon.

[42] Osswald S., Havel M., Mochalin V., Yushin G., Gogotsi Y. (2008). Increase of Nanodiamond Crystal Size by Selective Oxidation. Diam. Relat. Mater..

[43] Pasternak D.G., Romshin A.M., Bagramov R.H., Galimov A.I., Toropov A.A., Kalashnikov D.A., Leong V., Satanin A.M., Kudryavtsev O.S., Gritsienko A.V. (2024). Donor–Acceptor Recombination Emission in Hydrogen-Terminated Nanodiamond. Adv. Quantum Technol..

[44] Dexter D.L., Schulman J.H. (1954). Theory of Concentration Quenching in Inorganic Phosphors. J. Chem. Phys..

[45] Crossfield M.D., Davies G., Collins A.T., Lightowlers E.C. (1974). The Role of Defect Interactions in Reducing the Decay Time of H3 Luminescence in Diamond. J. Phys. C Solid State Phys..

[46] Singh S., Catledge S.A. (2013). Silicon Vacancy Color Center Photoluminescence Enhancement in Nanodiamond Particles by Isolated Substitutional Nitrogen on {100} Surfaces. J. Appl. Phys..

[47] Maier F., Riedel M., Mantel B., Ristein J., Ley L. (2000). Origin of Surface Conductivity in Diamond. Phys. Rev. Lett..

[48] Luo Y.-R. (2007). Comprehensive Handbook of Chemical Bond Energies.

[49] Ferrari A.C., Robertson J. (2000). Interpretation of Raman Spectra of Disordered and Amorphous Carbon. Phys. Rev. B.

